# Templated tibial plateau leveling osteotomy and implant guidance system improves accuracy in novice surgeons: an *ex vivo* study

**DOI:** 10.3389/fvets.2026.1729787

**Published:** 2026-01-29

**Authors:** Tyler J. Wyatt, Albert C. Lynch, Brianna Miniter

**Affiliations:** 1 A.C.L. Surgery, Philadelphia, PA, United States; 2 BluePearl Pet Hospital, Clearwater, FL, United States

**Keywords:** canine, conformation specific, cranial cruciate ligament (CCL), guidance system, three-dimensional modeling, tibial plateau leveling osteotomy (TPLO)

## Abstract

**Objective:**

Introduce and compare the accuracy of a templated tibial plateau leveling osteotomy and implant guidance system (ProCut TPLO) to the traditional tibial plateau leveling osteotomy (TPLO) in novice surgeons.

**Methods:**

Ten participants without osteotomy experience completed a TPLO (*n* = 10) and ProCut TPLO (*n* = 10) using canine cadaveric limbs. Deviation from intended tibial plateau angle (TPA), distance of eccentricity (DOE), osteotomy trajectory along proximodistal and craniocaudal axes, procedure time, and technical errors were compared. For ProCut TPLO limbs, a trajectory indicating pin was compared to the executed osteotomy trajectory along proximodistal and craniocaudal axes.

**Results:**

ProCut TPLO resulted in TPAs that deviated less from target (0.36° vs. 5.6°, *p* = 0.001), a DOE that was more centered (0.8 mm vs. 7.8 mm, p = 0.001), an osteotomy that was more parallel to joint surface (1.5° vs. 4.4°, *p* = 0.009) and perpendicular to sagittal plane (2.6° vs. 8.4°, *p* = 0.002), and took less time (23.1 min vs. 40.9 min, *p* = 0.005). Mean proximodistal and craniocaudal trajectory deviation from the ProCut TPLO trajectory indicating pin was 0.3° and 0.4°, respectively. No technical errors occurred with ProCut TPLO.

**Conclusion:**

The ProCut TPLO yielded more accurate technical execution across all metrics compared to the traditional TPLO and thus may offer novices an accurate and efficient method of executing TPLOs.

## 1 Introduction

Cranial cruciate ligament (CCL) injury is a common cause of joint instability and osteoarthritis (1, 2). The traditional tibial plateau leveling osteotomy (TPLO) is a widely used and effective treatment for CCL disease in dogs; however, it presents technical challenges, especially for novice surgeons (3, 4).

The TPLO involves performing a proximal tibial radial osteotomy to reduce the tibial plateau angle (TPA) and neutralize cranial tibiofemoral shear force (5). Executing the ideal radial osteotomy can be challenging for novices, especially in achieving precise centering and trajectory (3, 4). Jigs and guides have been developed to aid the execution of radial osteotomy, but their practical benefits remain unclear (3, 6–10). Use of patient-specific osteotomy and drill guides has been described, but their utilization can be limited by the need for CT imaging, computer modeling, and 3D printing (7, 8).

The TPLO procedure also requires precise execution of fixed plane rotation, temporary stabilization of post-osteotomy tibial bone segments, and accurate positioning of the bone plate and screws for definitive fixation (5). Studies suggest that cranial tibiofemoral shear force is neutralized at a TPA of 6.5° (11); however, even with board-certified surgeons, the post-operative TPA can vary markedly (4, 12).

In attempts to address the challenges described above, the ProCut TPLO (p-TPLO) system was developed. The p-TPLO is a guided and templated surgical procedure with an associated implant system designed to simplify the initiation and execution of the radial osteotomy, facilitate fixed plane rotation and compression of tibial bone segments, and ensure accurate implant positioning. The technique negates the requirement for manual alignment and temporary stabilization of the post-osteotomy bone segments. The authors hypothesize that novice surgeons using the p-TPLO system will achieve more accurate technical execution outcomes compared to the traditional TPLO technique.

## 2 Materials and methods

### 2.1 Study design

In this study, technical execution metrics were compared between the traditional TPLO and p-TPLO technique using veterinarians and veterinary students (*n* = 10) that had not performed any portion of an osteotomy previously. To rehearse the technical steps, participants performed a TPLO and a p-TPLO on a synthetic canine tibia bone model (Saw-bones, 2,117–20, Pacific Research Laboratories, Washington) (*n* = 20) using the radial saw blade (21 mm or 24 mm radial TPLO blade, Movora, St. Augustine, FL, USA) intended to be used on their cadaveric limbs. Data from rehearsal procedures were not evaluated.

Each participant then performed a TPLO and a p-TPLO on cadaver pelvic limbs from skeletally normal dogs (Skulls Unlimited, Oklahoma City, OK, USA) (*n* = 20) weighing 27–37 kg. The cadaveric limbs, euthanized for reasons unrelated to this study, showed no evidence of prior stifle pathology on palpation or radiographs. Participants were randomly assigned to paired limbs of the same animal to limit variability. Within each pair, treatment groups (TPLO and p-TPLO) and order of completion were randomly assigned to each limb using computer-generated randomization. The corresponding author performed a standard surgical approach to expose the proximal medial tibia and mark the joint surface using two 25-gauge hypodermic needles for consistency between groups. All subsequent steps were completed by participants, focusing on technical execution metrics. Technique guides were provided to participants during the experiment.

All radiographs were obtained using a single calibrated digital radiography system (Patterson RadMedix Acuity X-Ray System, Loveland, CO, USA). To minimize known sources of TPA measurement variability related to limb positioning (13), all images were performed by the same operator using standardized positioning criteria. Lateral radiographs were acquired with the stifle and tarsus held in 90° of flexion and with superimposition of the femoral condyles used as the criterion for acceptable positioning. Craniocaudal radiographs were obtained with the limb in extension, ensuring that the femoral cortices bisected the fabellae and that the medial aspect of the calcaneus bisected the tibiotarsal joint. All postoperative radiographs were performed immediately following completion of the procedure to maintain consistency in positioning and reduce temporal variability.

Pre-operative planning and post-operative technical execution measurements were obtained using surgical planning software (vPOP-Pro, Shropshire, GB). Three total measurement sets were performed by two board-certified surgeons, both experienced in the TPLO procedure but unfamiliar with the p-TPLO system and blinded to the study. One surgeon performed two measurement sets to assess intraobserver variability, and the second surgeon performed one set to assess interobserver variability. Accuracy was defined as the deviation between planned and achieved outcomes, described below. Technical errors, described below, were categorically assessed by the corresponding author.

### 2.2 Traditional TPLO technique

**Figure 1:**
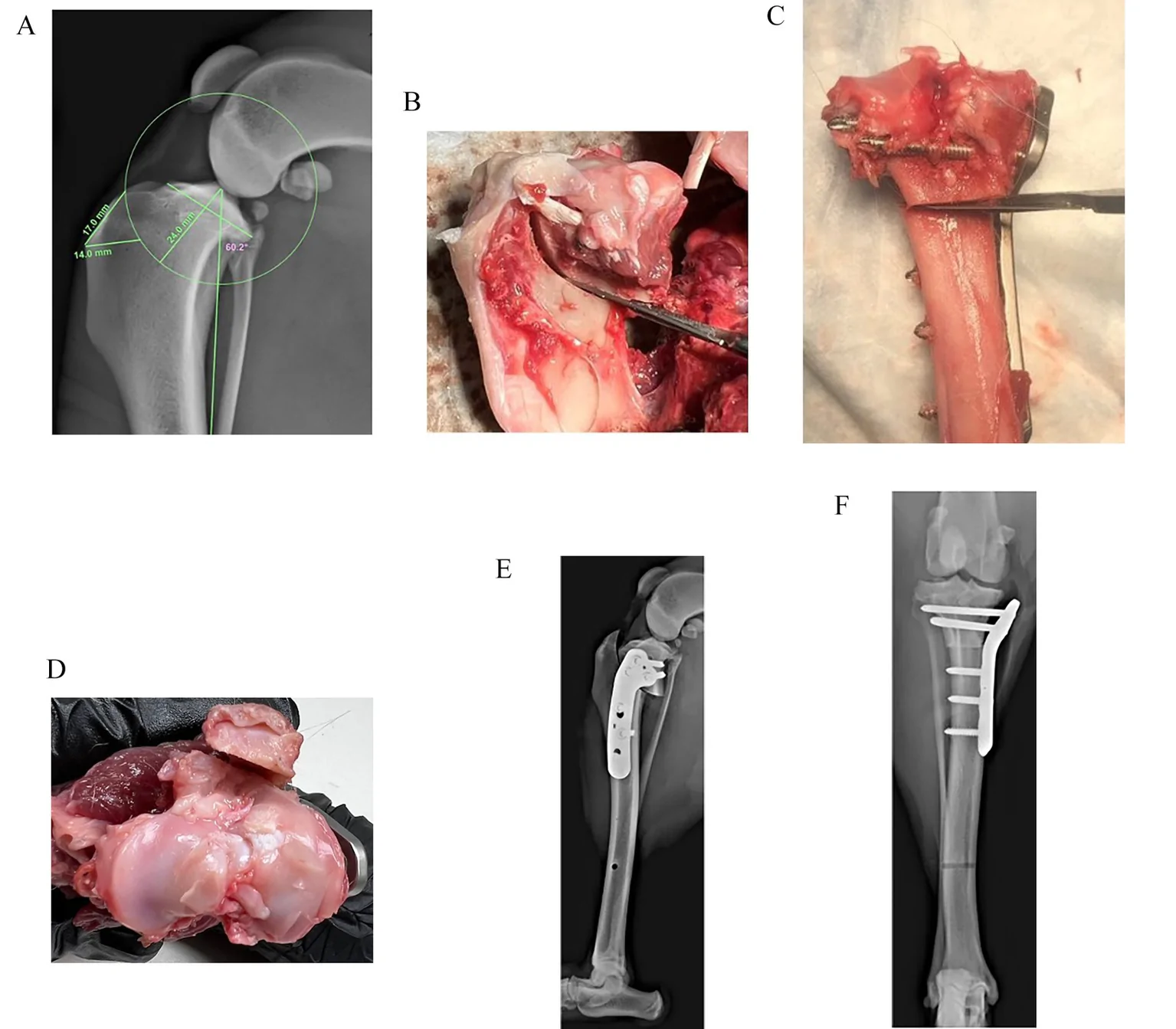
Representative traditional TPLO outcomes and technical errors. **(A)** Preoperative, calibrated radiographs are taken to determine tibial plateau angle (TPA), saw blade size, desired implant, and D1/D2 measurements. **(B)** Lateral aspect of proximal tibia revealing an osteotomy gap at the trans cortex. **(C)** Caudal aspect of proximal tibia revealing an osteotomy gap present at the caudal cortex and a screw present in the caudal popliteal fossa, with minimal bone purchase. **(D)** Coronal view of tibia demonstrating excessive cranial osteotomy trajectory in the craniocaudal axis. **(E)** Sagittal and **(F)** frontal plane postoperative radiographs.

Preoperative calibrated radiographs were taken to determine desired saw blade, D1/D2 measurements (14), TPA, and the implant (Versiv TPLO plate, Movora, St. Augustine, FL, USA) (Figure 1A), with a target postoperative TPA of 5°.

A Slocum-style TPLO jig (Movora, St. Augustine, FL, USA) was placed using 2.8 mm Steinmann pins, and D1/D2 measurements were marked on the tibial cortex with a fine-tip marker (Sharpie, Newell Brands, Atlanta, GA, USA). The radial osteotomy was partially executed, rotation marks made, and osteotomy was completed. A 2.8 mm Steinmann pin was inserted as a rotation pin, and a 1.6 mm temporary stabilization k-wire was used once the desired rotation was achieved. The jig, jig pins, and rotation pin were removed. Definitive fixation was temporarily positioned on the proximal and distal tibial segments using a 1.1 mm k-wire through the designated pin hole in the plate. The proximal tibial segment was secured with two 3.5 mm locking screws, avoiding the temporary stabilization k-wire. A 3.5 mm non-locking screw was placed in load in the distal tibial segment to compress the osteotomy. The remaining two distal screw holes were filled with 3.5 mm locking screws, after which the temporary stabilization k-wire was removed. The remaining screw hole in the proximal tibial fragment was filled with a 3.5 mm locking screw.

### 2.3 ProCut TPLO technique and implant system

**Figure 2:**
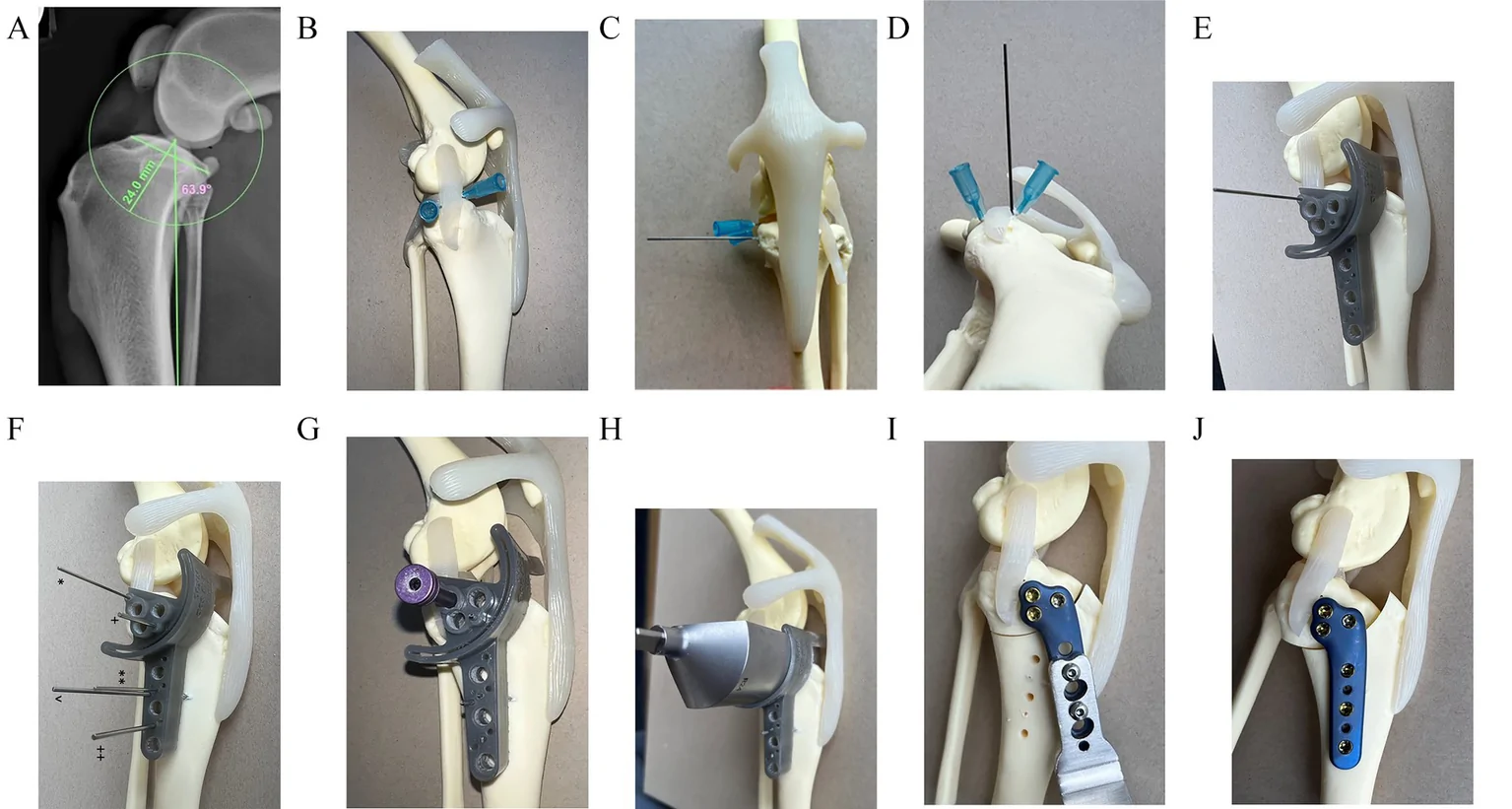
ProCut TPLO surgical technique. **(A)** Preoperative, calibrated radiographs are taken to determine tibial plateau angle (TPA) and p-TPLO template size. D1/D2 measurements are not required. **(B)** Step 1: Stifle joint surface is delineated with hypodermic needles after making standard medial approach to the proximal tibia. **(C)** Step 2: The 1.0 mm k-wire [proximal centering pin (PCP)] is placed 2–3 mm distal to the joint surface, approximately 3 mm into the proximal tibia just cranial to the medial collateral ligament. The PCP represents desired osteotomy trajectory and is assessed in the frontal and **(D)** coronal plane. Trajectory can be modified if needed by bending the PCP at its junction with the tibial cortex. **(E)** Step 3: Position the p-TPLO template on the PCP with the patellar tendon shield caudal to the patellar tendon. **(F)** Step 4: Once the template is positioned on the PCP (*), a second 1.0 mm k-wire (distal centering pin [DCP]) (**) is placed to center the distal aspect of the template and trajectory is again assessed. 1.6 mm proximal (^+^) and distal (^++^) temporary fixation pins are then placed, followed by a non-parallel fixation pin (^) to fix the desired trajectory. **(G)** Step 5: Using the p-TPLO drill sleeve in the template drill holes, drill all six screw holes using 3.0 mm bit. **(H)** Step 6: Execute radial osteotomy via the saw blade slot. **(I)** Step 7: The template, PCP, DCP, and distal pins are removed. During proximal screw insertion, the bone plate with rotation handle attached was manually held over the piloted screw holes, and all proximal Morse taper locking screws were inserted until initial contact with the bone plate, then sequentially tightened, pulling the plate flush to the bone, facilitating compression. **(J)** Step 8: Rotate the proximal bone segment until the piloted distal screw holes are aligned with the holes of the bone plate, then insert and tighten distal screws and detach rotation handle.

The p-TPLO technique and implant system [the latter of which was manufactured using titanium (Ti-6Al-4 V ELI)], were developed by the corresponding author. Preoperative, calibrated radiographs were performed to determine the desired saw blade and TPA (Figure 2A) (15). The p-TPLO templates for this study, specific to the radial blade size (10 mm – 30 mm radius), were manufactured using surgical guide V1 resin (Formlabs, Somerville, MA). These templates come in 2.5° increments from 15° to 30°, corresponding to the intended tibial plateau rotation needed to achieve a postoperative TPA nearest 5° (preoperative TPA − template degree rotation = target TPA). This range of angle corrections aims to capture the majority of clinically encountered TPAs.

After the stifle joint surface is delineated with hypodermic needles (Figure 2B), the proximal centering pin (PCP) and distal centering pin (DCP), both 1.0 mm smooth trocar tip pins, were placed to center the p-TPLO template and assess osteotomy trajectory (Figures 2C–E). Temporary fixation pins, 1.6 mm smooth trocar tip pins, were placed to secure the template (Figure 2F). All screw holes were then drilled with a 3.0 mm drill bit, osteotomy performed, pins and template removed, and the p-TPLO bone plate attached proximally using three 4.0 mm morse taper locking screws (Figures 2G–I). The proximal temporary fixation pin was initially placed bicortically and cut flush to the tibial cortex, rather than removed, to evaluate the executed osteotomy trajectory. The p-TPLO bone plate was rotated and attached distally using three 4.0 mm morse taper locking screws (Figure 2J).

### 2.4 Technical execution metrics and errors

For both the TPLO and the p-TPLO, technical execution metrics included absolute deviation of observed magnitude of tibial plateau sagittal plane rotation from expected, distance of eccentricity (DOE), osteotomy trajectory along the proximodistal and craniocaudal axes relative to the joint surface (16), time, and technical errors. Additionally, for p-TPLO limbs, the system trajectory indicating pin cut flush to the bone was used to evaluate the executed osteotomy trajectory along the proximodistal and craniocaudal axes relative to the pin.

To evaluate the TPLO and p-TPLO osteotomy trajectory along the craniocaudal axis relative to the joint surface, and to assess technical errors, the proximal tibia was disarticulated and dissected free of soft tissue. The tibial plateau was photographed (iPhone 13, Apple Inc., Cupertino, CA, USA) so that the osteotomy trajectory could be measured relative to the caudal margins of the tibial condyles.

To evaluate the p-TPLO osteotomy trajectory along the craniocaudal axis relative to the p-TPLO temporary fixation trajectory indicating pin, the dissected tibia was sectioned distal to the bone plate using a sagittal saw (Acculan 4 Mini Drill, Aesculap, Center Valley, PA, USA). The sectioned tibia was radiographed in the coronal plane so that the osteotomy trajectory could be measured relative to the p-TPLO fixation pin trajectory.

Major technical errors included loss of load sharing with the tibial bone segments, narrowed tibial crest with presence of an isthmus, loss of bone engagement by a bone screw, and joint penetration by a bone screw or jig pin. Load sharing was defined as lost if gaps were present at the trans and caudal cortices. Minor technical errors were those that represented deviation from the intended surgical technique or could reasonably lead to a clinical complication.

### 2.5 Statistical analysis

Statistical analysis was conducted using statistical software (R Version 2023.06.0 + 421). Because limited prior data exist on TPLO accuracy in novice surgeons, an *a priori* analysis was not performed; sample size was determined by cadaver and participant availability. Intra- and interobserver reliability were assessed using the intraclass correlation coefficient (ICC) calculated with a two-way mixed-effects model for absolute agreement of single measurements. The ICC ≤ 0.50 was considered poor reliability, between 0.5 and 0.75 as moderate reliability, between 0.75 and 0.9 as good reliability, and ≥0.9 as excellent reliability (17). Participants completed both procedures, generating paired data. Descriptive statistics, including mean, range, and standard deviation (SD) were computed. Outcomes were compared between TPLO and p-TPLO using Wilcoxon signed-rank tests, with statistical significance defined as a *p*-value < 0.05. The proximodistal and craniocaudal trajectory relative to the joint surface and relative to the proximal p-TPLO fixation pin were defined as previously described (16) and were converted into absolute values for statistical comparison accuracy.

## 3 Results

In all comparative metrics, the intra- and interobserver reproducibility showed excellent reliability (ICC range: 0.90–0.97, *p* < 0.001). As such, these outcome metric comparisons were performed with the mean values of the observers. Intraobserver reliability for the trajectory-indicating p-TPLO fixation pin metrics was poor for proximodistal deviation (ICC = −0.06, *p* = 0.60) and moderate for craniocaudal deviation. (ICC = 0.70, *p* = 0.01), while interobserver reliability was poor for both proximodistal (ICC = 0.02, *p* = 0.50) and craniocaudal deviation (ICC = 0.20, *p* = 0.30).

### 3.1 Tibial plateau angle

The mean preoperative TPA was 27.6° (range, 21–36) for all cadavers, with no difference (*p* = 0.87) between the traditional TPLO group (27.1° ± 3.7, range 21–34) and p-TPLO group (28.0° ± 4.5, range 23–36). Procedures performed with the p-TPLO resulted in postoperative TPAs that were closer to the target TPA than procedures performed using the traditional TPLO method (Table 1).

**Table 1:** Comparative technical execution metrics.

Execution Metric	Traditional TPLO	ProCut TPLO	*p*
TPA deviation (mean ± SD)	5.6 ± 1.9	0.36 ± 0.5	0.001
DOE (mean ± SD mm)	7.8 ± 1.8	0.8 ± 0.8	0.001
PD trajectory deviation from joint (mean ± SD)	4.4 ± 3.7	1.5 ± 1.61	0.009
CC Trajectory deviation from joint (mean ± SD)	8.4 ± 3.8	2.6 ± 2.3	0.002
Time (mean ± SD min)	40.9 ± 5.8	23.1 ± 2.4	0.005

CC, craniocaudal; DOE, distance of eccentricity; PD, proximodistal; SD, standard deviation; TPA, tibial plateau angle; TPLO, tibial plateau leveling osteotomy.

### 3.2 Distance of eccentricity

**Figure 3:**
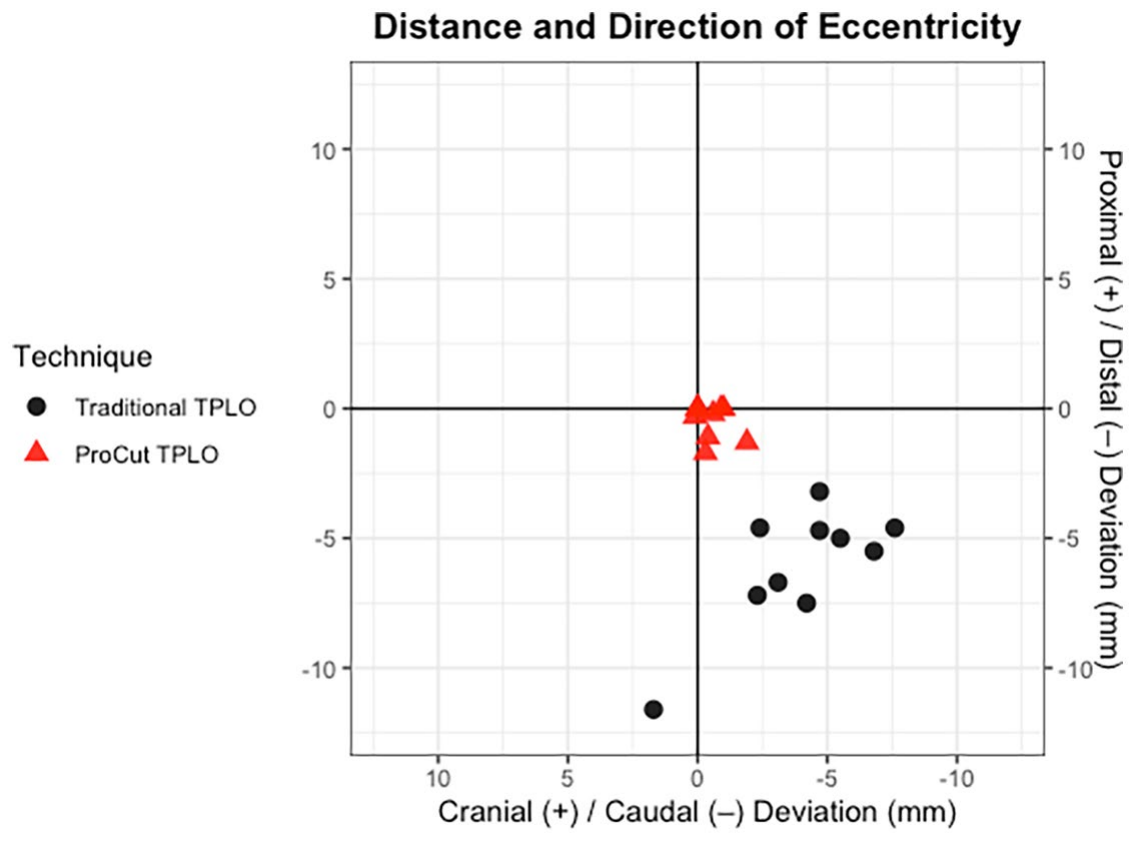
Distance and direction of eccentricity. Distance and direction of osteotomy eccentricity for procedures performed using the traditional TPLO technique (black circles) and the ProCut TPLO system (red triangles). The *x*- and *y*-axes intersect at the intended centroid of the osteotomy. Each data point represents the actual centroid achieved by the participant. Axes are arranged to reflect the standard orientation of a hanging stifle radiograph. Positive *x*-values represent cranial deviation and negative values represent caudal deviation, while positive *y*-values represent proximal deviation and negative values represent distal deviation.

Osteotomy performed with the p-TPLO resulted in a saw blade that was more centered around the intercondylar eminence than the traditional TPLO (Table 1 and Figure 3).

### 3.3 Proximodistal trajectory relative to joint

Osteotomy performed with the p-TPLO system resulted in an osteotomy that was more parallel to the tibial plateau than the traditional TPLO (Table 1).

### 3.4 Craniocaudal trajectory relative to joint

Osteotomy performed with the p-TPLO system resulted in an osteotomy that was more perpendicular to the sagittal plane than the traditional TPLO (Table 1).

### 3.5 Proximodistal trajectory relative to p-TPLO fixation pin

The mean proximodistal trajectory deviation relative to the p-TPLO proximal fixation pin was 0.3° ± 0.2 (range, 0.1–0.4).

### 3.6 Craniocaudal trajectory relative to p-TPLO fixation pin

The mean craniocaudal trajectory deviation relative to the p-TPLO proximal fixation pin was 0.4° ± 0.4 (range, 0.1–1.4).

### 3.7 Time

Procedures performed with the p-TPLO system took less time than those performed using the traditional TPLO method (Table 1).

### 3.8 Technical errors

The p-TPLO procedures resulted in no major or minor technical errors. The traditional TPLO procedures resulted in both major and minor technical errors. Major errors observed included osteotomy gaps that result in loss of load sharing, loss of functional screw purchase, and a narrowed tibial crest with the presence of an isthmus. Load sharing loss occurred in 3/10 TPLO procedures (Figures 1B,C). Loss of functional screw purchase occurred in 7/10 TPLO procedures with screws being placed in the caudal popliteal tibial fossa (Figure 1C). In each case, this involved a single proximal caudal screw with varying degrees of monocortical engagement of the medial tibial condyle. A narrowed tibial crest with the presence of an isthmus occurred in 1/10 TPLO procedures. Minor errors observed in the TPLO procedures included a double osteotomy cut of the proximal medial tibial cortex, which occurred in 1/10 TPLO procedures.

## 4 Discussion

It is suggested that approximately 50 traditional TPLO procedures are needed to become proficient in osteotomy positioning and trajectory (4). The p-TPLO system was developed as a fully guided and templated surgical procedure to address the technical challenges of a TPLO procedure, particularly for novice surgeons. The present investigation found that the p-TPLO system improved technical execution and reduced technical errors, compared to the traditional TPLO, in novices performing the cadaveric TPLO in this study. It is important to note that results from this ex-vivo study cannot be directly extrapolated to the in-vivo clinical setting.

**Figure 4:**
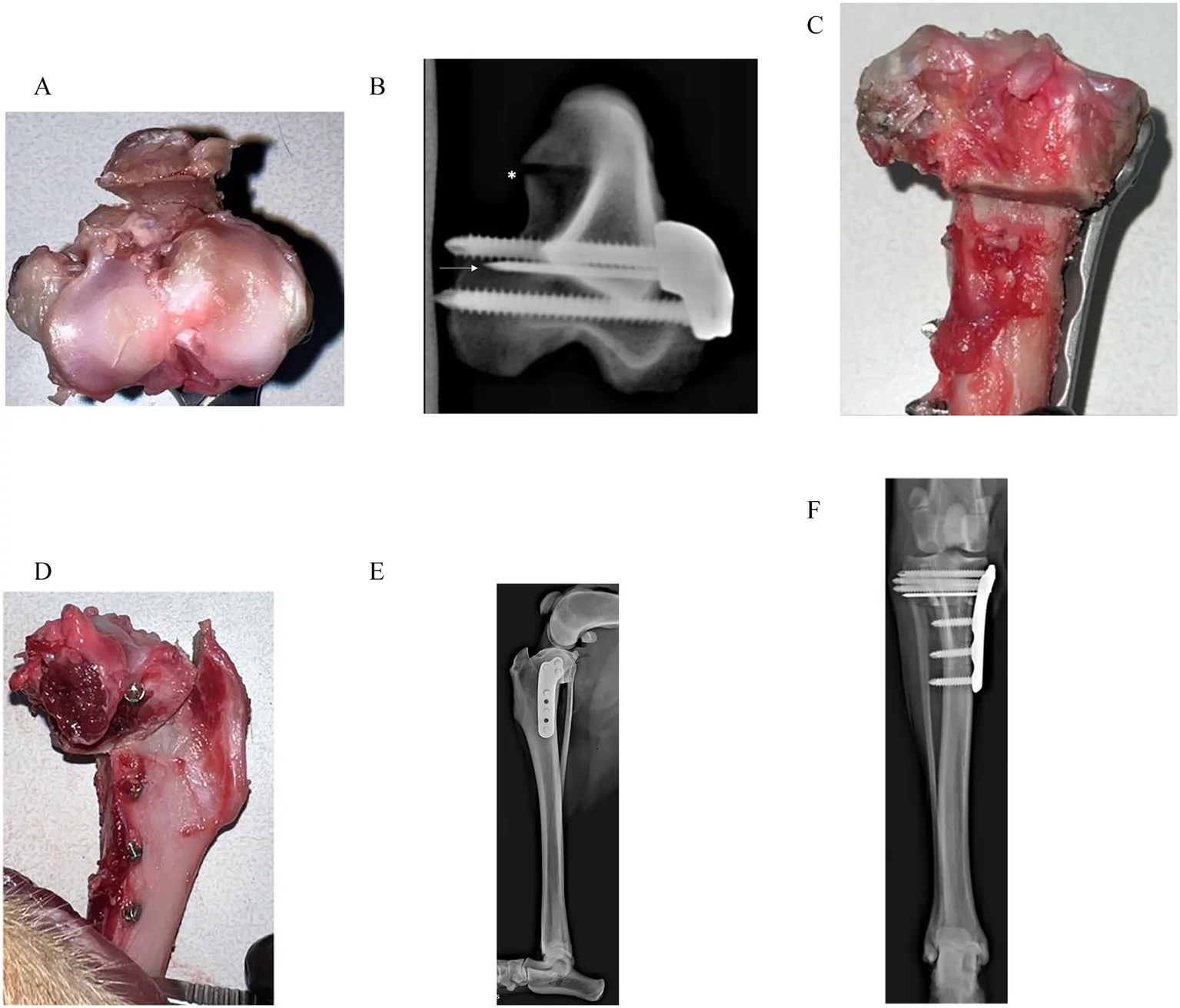
Representative ProCut TPLO outcomes. **(A)** Coronal view of tibia demonstrating the executed osteotomy trajectory in the craniocaudal axis. **(B)** Radiographic comparison of the executed osteotomy (*) to the p-TPLO proximal temporary fixation pin (arrow). **(C)** Caudal aspect of proximal tibia revealing appropriately compressed osteotomy and no screws present in the caudal popliteal fossa. **(D)** Lateral aspect of proximal tibia revealing appropriately compressed osteotomy. **(E)** Sagittal and **(F)** frontal plane postoperative radiographs.

In the assessment of osteotomy angulation, the p-TPLO group demonstrated significantly more accurate proximodistal and craniocaudal trajectories relative to the joint (Figures 1D,F, 4A). Previous studies have also reported the efficacy of three-dimensionally printed saw guides improving osteotomy angulation (7, 8, 10). An advantage unique to the p-TPLO is the ability to assess and alter the osteotomy trajectory in the frontal and coronal planes after initial pin placement but before temporary fixation of the template.

The p-TPLO osteotomy trajectory along the proximodistal and craniocaudal axes is influenced by the intraoperative assessment of the joint surface relative to the centering pins, as the p-TPLO template is designed to dictate the executed trajectory. This proved manageable, with mean deviations from ideal trajectory being 1.5° and 2.6° in the proximodistal and craniocaudal axes, respectively. These results are comparable to the osteotomy angulation achieved by TPLO-experienced surgeons, both using and not using a jig (18).

To assess accuracy of the executed p-TPLO osteotomy relative to its fixation pins, the proximal temporary fixation pin, representing the surgeon-defined trajectory, was cut flush to the tibial cortex instead of being removed. Radiographic comparison of the executed osteotomy to the pin revealed nearly identical trajectories in both axes, supporting the hypothesis (Figure 4B). The associated numerically low intra- and interobserver reliability values were anticipated and likely reflect the narrow range of observed values (all measurements within 1.4° of the guide trajectory), which results in minimal between-subject variance in a smaller sample size (17). Under these conditions, even small absolute measurement error can disproportionately depress correlation-based reliability metrics. Importantly, the absolute angular differences between measurements were small and likely to be clinically negligible.

Postoperative TPA is influenced by positioning of the osteotomy center, assessed by DOE. Previous studies of patient-specific osteotomy guides have not shown improved osteotomy centering compared to traditional methods (7, 8). The traditional TPLO group demonstrated predominantly caudodistal DOE, consistent with a prior report (12). This may reflect surgeons centering the cut relative to the proximal jig pin rather than the true intended osteotomy center or biasing the cut distally to maintain sufficient bone stock for plate application. In the p-TPLO, DOE is determined by the positioning of the PCP, which is intentionally placed eccentric to the true center of rotation to ensure safe, reproducible placement at a controlled distance from the joint surface and medial collateral ligament. The PCP’s location is standardized across specimens and is based on the final position of tibial bone segments, with its specific distocranial offset varying by blade size. This design maintains the intended center of rotation around the intercondylar eminence while allowing for precise and surgically accessible PCP placement, contributing to the reproducibility of the centered osteotomy and final tibial plateau angle adjustment. Here, the p-TPLO resulted in a significantly improved DOE and less deviation from the intended postoperative TPA (Figures 1E, 3, 4E). Notably, novices using the p-TPLO achieved a mean DOE and postoperative TPA closer to the target than those reported for experienced surgeons performing both free-hand (4) or jig-assisted (12) TPLO procedures, while recognizing that TPA variation is multifactorial and not soley attributable to DOE. All TPA measurements reported here were obtained without obstruction of necessary anatomical landmarks, further supporting the validity of the reported accuracy in the p-TPLO group.

Compared to the traditional TPLO group, the p-TPLO reduced procedure time and resulted in no major or minor technical errors (Figures 4A,C,D). The two most common major technical errors in the traditional TPLO group were the placement of a screw in the caudal popliteal fossa of the tibia and the presence of an osteotomy gap that led to loss of load sharing, both of which were difficult to identify during the cadaveric procedure and postoperative radiographs (Figures 1E,F). The unexpected finding of osteotomy gaps at the trans and caudal cortices, despite no indication during the procedure or on postoperative radiographs, warrants further investigation.

Improper screw placement, such as placing a screw in the caudal popliteal fossa of the tibia without engaging significant bone, can compromise fixation stability and increase the risk of implant failure and complications (19). This may result from improper plate placement or the specific TPLO plate design. The p-TPLO system may address this issue by improving plate placement, thereby reducing the risk of improperly placed screws. Unique to this technique, all six screw holes are drilled simultaneously after the drill-guide template is centered and temporarily fixated, but prior to execution of the radial osteotomy. The proximal screw holes are rotationally offset from the distal ones, aligning the tibia to the intended TPA once the p-TPLO bone plate is applied. Unlike the traditional TPLO, the p-TPLO does not require rotation pins or temporary stabilization k-wires. Instead, a handle attached to the p-TPLO bone plate, anchored to the proximal post-osteotomy bone segment via the proximal screws, allows the surgeon to rotate the tibial plateau in a fixed plane, aligning the distal screw holes with the pre-piloted holes in the tibial diaphysis.

In the traditional TPLO group, 9 out of 10 cadaveric procedures had an osteotomy gap at one or more cortices, with 3 out of 10 exhibiting loss of load sharing. This unintended failure to achieve load sharing can increase the risk of implant failure and prolong healing times (20). In contrast, no osteotomy gaps were observed in the p-TPLO group. This may be explained by the p-TPLO system’s design, which aims to create interfragmentary compression through a combination of axial and angular mechanisms. Templates account for saw kerf loss and deliver axial compression. Distal screw holes are pre-piloted distocranially relative to the final locking position to ease placement and encourage trans-osteotomy contact. Morse taper locking screws facilitate these compressive forces by progressively engaging the plate upon final locking.

The authors identified several limitations to this investigation. First, study participants may not fully represent the broader population of novice surgeons learning the TPLO procedure and do not include experienced TPLO surgeons. Our goal was to evaluate the accuracy of the p-TPLO system in inexperienced hands compared to the traditional TPLO technique. Future studies comparing experienced TPLO surgeons using both techniques are warranted. Second, as a cadaveric study with limited sample size and unknown breed and age distribution, it does not fully represent in-vivo conditions. Whether the same outcomes would be observed in patients with naturally occurring cranial cruciate ligament disease remains to be seen. Third, the use of a Sharpie marker on cadaver specimens made a subjectively wide mark, potentially affecting the accuracy of tibial plateau rotation for the traditional TPLO group. Future studies might achieve a narrower post-operative TPA range using an osteotome or measuring the caudal overhang. Fourth, only a single prototype p-TPLO implant size was available for this study, which resulted in occasional plate undersizing relative to specimen tibial dimensions but does not reflect constraints imposed by the p-TPLO system. As the focus of this investigation was technical accuracy rather than mechanical performance, all specimens were instrumented with the same implant size to limit variability. Lastly, though the board-certified surgeons performing the measurements in this experiment were blinded to the study and unfamiliar to the p-TPLO system, no attempts were made to obscure potentially distinguishable features such as jig pin holes, k-wire holes, or plate design so as not to interfere with measurements.

The authors also acknowledge that the p-TPLO system described here requires multiple printed templates per blade size, which would represent a practical limitation if translated directly into clinical use. The system has since undergone further development to reduce template inventory requirements. In the production-intended design, each blade size corresponds to a specific plate size to optimize proximal screw purchase and bone stock, and a reusable metal template system is being prototyped. This system consists of a left and right main template body with fixed guides, used in conjunction with interchangeable, reversible faceplates that provide the required rotational offsets in 2.5° increments. This modular configuration reduces the overall number of components needed while maintaining drill-guide precision. Although detailed engineering evaluation is beyond the scope of this study, this developmental direction addresses several logistical considerations relevant to future clinical application.

In conclusion, we documented that the p-TPLO resulted in more accurate technical execution metrics and reduced technical errors compared to the traditional TPLO in novice surgeons performing TPLO procedures in this cadaveric group. Based on these findings, evaluation of the system with surgeons experienced in TPLO surgery with potential extrapolation to clinical use could be considered.

## Data Availability

The raw data supporting the conclusions of this article will be made available by the authors, without undue reservation.
