# Correction: Templated tibial plateau leveling osteotomy and implant guidance system improves accuracy in novice surgeons: an *ex vivo* study

**DOI:** 10.3389/fvets.2026.1975703

**Published:** 2026-09-15

**Authors:** Tyler J. Wyatt, Albert C. Lynch, Brianna Miniter

**Affiliations:** 1 A.C.L. Surgery, Philadelphia, PA, United States; 2 BluePearl Pet Hospital, Clearwater, FL, United States

**Keywords:** canine, conformation specific, cranial cruciate ligament (CCL), guidance system, three-dimensional modeling, tibial plateau leveling osteotomy (TPLO)

The conflict of interest statement was erroneously given as:

The author(s) declared that this work was conducted in the absence of any commercial or financial relationships that could be construed as a potential conflict of interest.

The correct **Conflict of interest** statement is:

AL is the inventor of the p-TPLO (ProCut) system evaluated in this study and is the named inventor on pending patent applications covering the technology and intellectual property and is disclosed as a potential competing interest.

The remaining author(s) declared that this work was conducted in the absence of any commercial or financial relationships that could be construed as a potential conflict of interest.

The original version of this article has been updated.

